# Development of a Theory-Based Adolescent Health Literacy Intervention: A Case of Nepal

**DOI:** 10.3928/24748307-20260413-01

**Published:** 2026-07

**Authors:** Shanti Prasad Khanal, Chitra Bahadur Budhathoki, Bhimsen Devkota, Shyam Krishna Maharjan, Orkan Okan

**Affiliations:** a Faculty of Education, Tribhuvan University, Kathmandu, Nepal;; b Technical University, Munich, School of Medicine and Health, Center for Health and Medicine in Society, Center for Health Promotion in Childhood and Adolescence, Munich, Germany.

## Abstract

**Background:**

Evidence indicates a lack of school-based interventions targeting adolescents' health literacy. To date, no structured, theory-driven, Delphi-guided health literacy intervention has been designed for Nepalese adolescents.

**Objective:**

This study aims to report the systematic development of a context-specific intervention to enhance adolescent health literacy and health-promoting actions.

**Methods:**

Using a concurrent mixed-methods design and a comprehensive health literacy model, the research involved 501 9th grade students from four randomly selected community schools in Birendranagar Municipality, Surkhet District, as well as their health and physical education teachers (HPE) and school nurses. Quantitative data were collected using self-administered standardized questionnaires measuring health literacy, self-efficacy, perceived social support, school environment, community environment, and health-promoting actions. Qualitative data were obtained through focus group discussions with students and key informant interviews with HPE teachers and school nurses.

**Key Results:**

The intervention design process followed the fundamental steps of the intervention mapping approach. After engaging with the school through formal and informal means, health literacy needs were identified and prioritized. Goals were then set, focusing on the individual and interpersonal levels. Next, all components of the intervention, including objectives, content, facilitators, methods, materials, modes, and evaluation techniques, were refined through a two-round modified Delphi process, resulting in the first draft. This draft was discussed with students, teachers, and school nurses before finalizing. The completed intervention, consisting of 13 sessions, is designed to be implemented by nine interdisciplinary facilitators using the SHOWED questioning framework (What do you see here? What is happening? How does this relate to our lives? Why does the problem exist? What can we do?).

**Conclusions:**

This article reports on an intervention development process, systematically using mixed methods and the Delphi process, to offer a replicable approach to enhance adolescent health literacy and health-promoting actions.

## Abstract (plain language summary)

**Plain Language Summary: **We developed a school health literacy program, using the comprehensive model of health literacy to improve the school adolescents' ability to handle health information and health activities. A hard mixed-methods design identified the HL needs, and a modified Delphi study was used to refine the main components of the intervention. This development process resulted in a total of 13 sessions. The sessions were planned to be carried out using a dialogical pedagogy by facilitators from different interrelated areas in the schools. The program provides schools with a simple, cheap, and suitable method of educating adolescents on health literacy and health-promoting actions. It provides a realistic example, which can be adopted by other schools.

Health literacy (HL) focuses on maintaining a healthy lifestyle, understanding health determinants, and modifying them to enhance one's, families', and community's health (Kanj & Mitic, 2009). To achieve this, an individual's ability to access, understand, appraise, and apply health information to make informed judgments and decisions in everyday life regarding health care, disease prevention, and health promotion is at the core of the concept (Sørensen et al., 2012). While it has always been viewed as holistic when conceptualizing HL from a public health perspective (Casteleijn, 2016). The organizational HL (OHL) concept emerged around a decade ago, focusing on reducing the barriers, demands, and complexities that individuals face when interacting with health care and other organizations (Santana et al., 2021). OHL has been adapted to educational contexts through the Health Literate School framework (Kirchhoff et al., 2022; Kirchhoff et al., 2025) and the Health Literate Pre-School framework (Rauschmayr et al., 2025). Adolescent health literacy (AHL) is gaining attention (Riiser et al., 2020), although it is often viewed through the lens of adult HL (Bröder et al., 2017; Gordon et al., 2011; Massey et al., 2012).

Health problems are more widespread in Karnali province (Dhimal et al., 2022). Adolescents in Karnali face higher rates of early marriage, teen pregnancy, limited access to family planning methods, nutritional issues, and the highest fertility rate (Ministry of Health and Population [Nepal] et al., 2022). Evidence from Birendranagar Municipality, Surkhet, shows that adolescent students in community schools have limited knowledge across various health areas, such as SRH knowledge (Sapkota et al., 2024), high level of undernutrition (Bhatta et al., 2024), and low HL levels (Khanal et al., 2024). A significant gap exists between HL and health-promoting actions (HPAs) among the youth of the community schools here. These findings emphasize the need for HL intervention to improve the situation (Khanal et al., 2021, 2023, 2024).

Despite several efforts made by the World Health Organization to emphasize the co-benefits of addressing HL in the education sector and the vital role of schools and teachers in promoting HL in school-aged children early in life (Okan et al., 2020; WHO, 2017). There are almost no school-based HL interventions, or those that exist are not practical (Nash et al., 2021).

Among the few theory-based HL interventions for adolescents, only a few report their extensive effectiveness. Such interventions include a problem-based learning HL intervention for girls founded on constructivist learning theory (Karimi et al., 2019), a social learning-based HL promotion intervention designed using Bandura's social learning theory (Begjani et al., 2024), and a physical activities-based school program developed with a comprehensive model of HL (Knisel et al., 2020). Therefore, interventions that combine theoretical foundations with experiential learning tend to produce changes across multiple dimensions. These interventions are developed through a structured approach, ensuring their effectiveness and feasibility (Morgado et al., 2022). Aside from those few studies indicating a positive association between child and adolescent HL and health outcomes, Theory-based HL interventions are generally scarce, particularly in low-income countries (Khanal et al., 2023), including Nepal.

Consequently, this intervention is designed using the comprehensive HL model. This model considers HL as individuals' ability to process information, providing a multidimensional approach and multiple factors for school HL interventions (Sørensen et al., 2012) and a dialogical learning approach. To address this gap, this study aims to develop systematically a theory-based school-based HL intervention for adolescents using the intervention mapping approach. So, this article reports only the systematic intervention design process, using mixed-methods needs identification, a theory-driven process, and a modified Delphi survey.

## Methods

### Study Design

**Figure 1.**
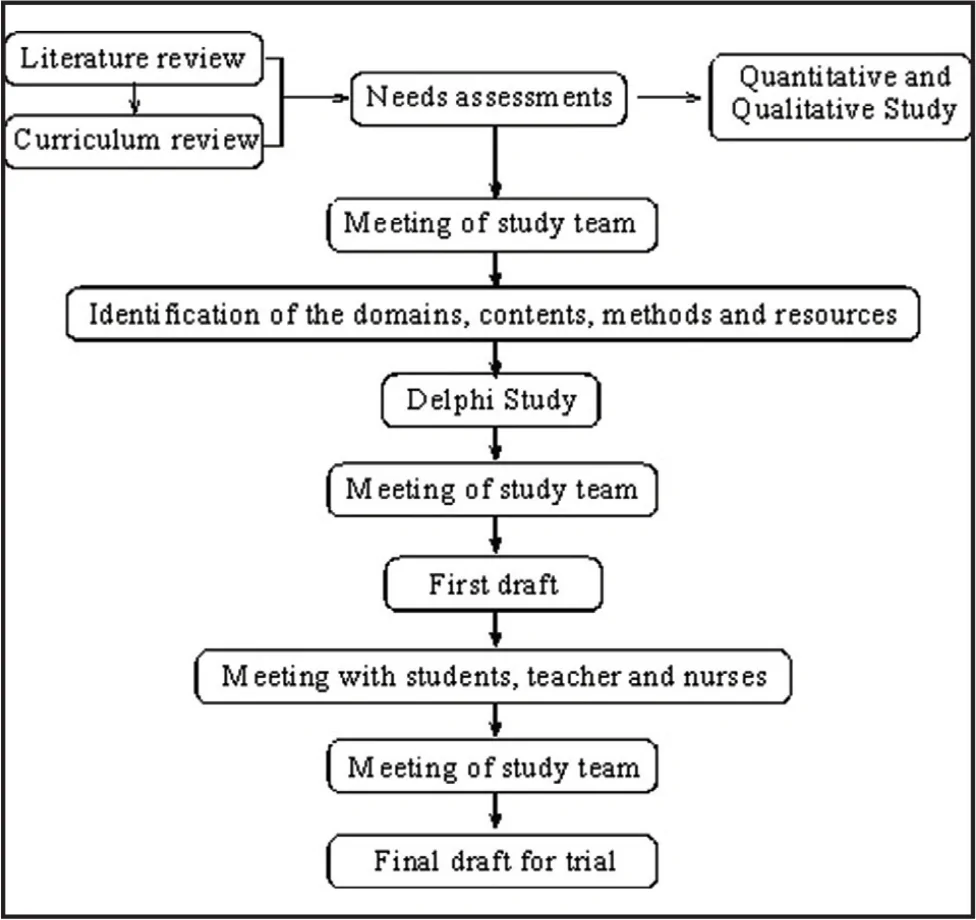
Overview of intervention development process.

This article outlines the intervention development process. We conducted a concurrent mixed-methods health literacy needs assessment to inform the intervention development (**Figure 1**).

### Study Setting and Sampling

This study was conducted in four randomly selected community schools located in Birendranagar Municipality, Surkhet District, Nepal. Schools were randomly selected to balance the representation of community school contexts in Birendranagar Municipality, reducing selection bias. The study population consisted of all ninth-grade students, their health and physical education teachers (HPE), and school nurses. Using standard sample size criteria for a cross-sectional study, *N* = 501 students were selected via census method for a quantitative needs assessment. Detailed information about sampling methods and calculations has already been reported (Khanal et al., 2025, 2026). Furthermore, 24 students, three HPE teachers, and four school nurses were purposively selected for the qualitative study.

### Data Collection Tools and Methods

Quantitative data were collected using various self-administered standardized scales. HL awareness was measured with a yes-or-no scale consisting of 25 items that addressed the meaning, benefits, consequences, and sources of health information, developed by the study team. The General Self-Efficacy Scale (GSES), a standardized scale, was used to measure students' overall self-efficacy (Schwarzer & Jerusalem, 1995). To examine the HL level of school-aged adolescents, the Nepalese-language version of the HLS-Child-24 NEP scale was used (Khanal et al., 2024), which was developed based on the original German questionnaire (Bollweg, Okan, Freţian, et al., 2020; Bollweg, Okan, Pinheiro, et al., 2020). This scale covers four dimensions: assessing (4 items), understanding (8 items), appraising (7 items), and applying (5 items) health information. These are summarized under one of the following three health domains: health care (8 items), disease prevention (8 items), and health promotion (8 items). Lastly, the study utilized the Adolescent Health Promoting-Short Form scale to examine school-aged adolescents' AHPs, including 21 items on nutrition (3 items), social support (4 items), health responsibility (4 items), life appreciation (4 items), exercise (4 items), and stress management (3 items). The AHP-SF is a valid and reliable tool for evaluating adolescent health-promoting actions, with a Cronbach's alpha of 0.90 and sub-scale ranges from 0.70 to 0.90 (Chen et al., 2014).

Additionally, structured questionnaires were pre-tested and refined to enhance consistency and reliability in the responses. Cronbach's alpha values were computed for scales demonstrating satisfactory to high internal consistency, including GSE (0.68), HLS-child-Q24-Nep (0.81), and the AHP-SF (0.70) (Khanal et al., 2025, 2026).

To gain in-depth qualitative insights, four focus group discussions (FGDs) were conducted with students, and seven key informant interviews (KIIs) were conducted with health and physical education teachers and school nurses. Four FGDs were conducted (Cresswell, 2012) with students to understand HL needs (Baumeister et al., 2021; Sørensen et al., 2012). The FGD guideline was prepared focusing on awareness, HL dimensions (access, understanding, appraisal, and application), three health domains (health care, disease prevention, health promotion), and health-promoting actions. Seven KIIs were conducted with HPE teachers and school nurses to explore the HL needs and to get feedback for the intervention. The KII guidelines were prepared addressing the HL situation, health-promoting actions, teaching methods, human resources, and intervention modes.

### Intervention Development Process

The development of the intervention followed a systematic approach grounded in the fundamental steps of the intervention mapping approach (Bartholomew et al., 2006). The process of designing the intervention started with understanding and engaging the target participants and context. Next, the process followed a mixed-methods needs assessment phase (Creswell & Creswell, 2018; Nastasi et al., 2007), which included a literature review (McKenna et al., 2025) to identify important domains and resources related to AHL. Considering this information, intervention outcomes and objectives were established, incorporating the comprehensive model of HL (Sørensen et al., 2012), and domains of the health-promoting actions (Chen et al., 2014). That guided the development of a theory-based intervention. This step was followed by a modified Delphi study to gain consensus on priority areas (Schneider et al., 2016). Then, an intervention draft was developed and reviewed iteratively (Schultes, 2023) after each meeting with stakeholders and subject matter experts. Comments from these reviews were used to further refine the intervention drafted, and the intervention was finalized for trial.

The intervention development phase adopted the SHOWED dialogical questioning framework as the pedagogical approach for delivery. This determination was informed by needs assessment findings such as the traditional way of health education teaching, adolescents' preference and, teachers' recommendations for interactive learning methods, and the Delphi panel's support for the use of a dialogical framework to foster active engagement and critical reflection. The SHOWED dialogical approach is based on Freirean critical pedagogy, incorporating listening, dialogue, and action, popularly used in participatory health education learning (Wilson et al., 2007). Such pedagogy would be used in HL education to develop students' HL skills in real-life situations (Sørensen et al., 2012). The intervention planned to adapt pedagogy to the school classroom context, incorporating the structure of guided discussions, small-group activities, and interactions.

### Delphi Process

Based on the needs analysis, a two-round modified Delphi approach was employed to gather consensus from panel members and validate the components of the intervention (Schneider et al., 2016). The relevance and clarity of the proposed components of the intervention were assessed by respondents using a 5-point Likert scale, with a total of 64 items. A total of 35 experts in the field of university faculty members, school health education teachers, school nurses, medical doctors, and public health officers were requested to participate; among them, 22 showed their interest. In Round 1, 22 experts were invited (**Table 1**), and 17 responded, resulting in a 77.27% response rate. They were also advised to provide qualitative comments and recommendations. Round 2 involved 15 experts, yielding a response rate of 88.24%. The anonymized summary of Round 1 feedback was provided to participants, and they were asked to re-rate items that had not reached consensus. The predetermined consensus was that an item should be considered concordant if at least 80% of the panel members rated it as *agree* or *strongly agree*. The items that failed to reach consensus after Round 2 were excluded from the final intervention based on relevance and feasibility (**Table 2**).

**Table 1:** Profiles and Disciplines of Delphi Panel Experts

**Personal Profile**	**Frequency**	**Percentage**

Sex		
Male	8	47.1
Female	9	52.9

Age (y)		
25–34	5	29.4
35–44	8	47.1
45–59	4	23.5

Working area		
University	5	29.4
School	7	41.2
Hospital	2	11.8
Public health	3	17.6

Job title		
Assistance professor	4	23.5
Medical doctor	2	11.8
School nurse	3	17.6
Public health officer	3	17.6
Teacher	4	23.5
Associate professor	1	5.9

Years of experience		
Below 10	10	58.8
11–20	4	23.5
More than 21	3	17.6

Qualification		
PhD	1	5.9
MD/MBBS	2	11.8
PCL nursing	3	17.6
Master's degree	5	29.4
Bachelor's degree	1	5.9
MPhil	5	29.4

Note. PCL = Proficiency Certificate Level.

**Table 2:** Summary of the Delphi Process and Consensus Items

**Round**	**Experts Invited**	**Expert Responded**	**Items Rated, %**	**Consented items**	**Items Revised**
Round 1	22	17	77.27	41	11
Round 2	17	15	88.24	11	13 excluded

The criteria for selecting panelists included a solid background and expertise in relevant health topics, current government-sector employment, at least 1 year of experience in a related field, and a strong interest and commitment to participating in this study.

### Data Analysis

The study employed descriptive statistics including frequency, mean, and standard deviation to analyze HL needs and guide intervention design; no effectiveness tests were performed. Delphi data were analyzed using the frequency and percentage of responses in the given categories. Consensus was determined based on 80% or more of the participants *agreeing* or *strongly agreeing*.

We analyzed qualitative data using a framework analysis approach (Ritchie & Spencer, 1994). Qualitative data were transcribed and translated, then reviewed several times to become familiar with the data. The principal author conducted coding in three rounds to enhance the analytical robustness (Locke et al., 2022), developing 50 codes, 12 categories, and five main themes. The themes were generated and linked to the theoretical framework used. To ensure the trustworthiness of the data, the study applied data triangulation, a validity procedure that combines multiple methods to form themes or categories (Creswell & Miller, 2000) as well as member checking (Creswell, 2012; Lincoln & Guba, 1985). The study integrated qualitative and quantitative data using a joint data display approach (Creswell & Creswell, 2018) to achieve a thorough understanding.

### Ethical Consideration

This study protocol has been approved by the Nepal Health Research Council (NHRC) (NHRC, Ref. #2688, date: 30 March 2022). Before participating in the study, parents provided written consent. The consent form, along with the participant information sheet, was sent home to the students and returned after the respective parents signed it. It was obtained with the class teacher's support. Students also provided their assent, and participation was entirely voluntary. Additionally, safeguarded are the participants' and the information's confidentiality.

## Results

The result section only deals with the outputs of the intervention design process, not with the implementation process or the effectiveness of the intervention.

### HL Need Assessment

A concurrent mixed-methods needs assessment process identified multifaceted needs to inform the design of the HL intervention. Quantitative results related to this have already been published (Khanal et al., 2026). Here, a summary of the key gaps, significant for intervention development, is integrated with qualitative insights (**Table 3**).

**Table 3:** Integrated Key Health Literacy Needs and Implications for Designing an Intervention

**Main Gaps**	**Quantitative Results**	**Qualitative Results**	**Design Implications**

Low HL awareness	Never heard (71.3%)	Unfamiliarity with the health literacy notion is common *Do not know. Heard it for the* *first time today* (FGD 1, ID 2, school 1)	HL introductory session
	Limited meaning of HL (74.7%)		
	Limited familiarity with the advantage of HL		
	Limited awareness of consequences of poor HL		

Self-efficacy	Low (33.5%), moderate (33.1%), high (33.3%)	-	-

Average HL level	Moderate (34.7%), low (31.7%)	HL is entirely poor	Skills in accessing, understanding, assessing, and applying health information
		From a practical point of view, their health literacy is weak (KII 1, school 1, school nurse)	

Low level across HL dimensions	Accessing (40.7%), assessing (39.5%), applying (38.5%), and understanding (33.5%)	Widespread difficulties in HL dimensions	-
		They do not use the health information they receive (KII, 3, school 3, HPE teacher)	

Difficulties in the health domains	Health promotion (37.7%), health care (36.3%) and disease prevention (32.8%)	Unfamiliar with the core concept of health promotion, the medical meaning of health care, and the unidimensional concept of disease Don't know anything about health promotion. Not even heard (ID 5, FGD 1, school 1)	Session on conceptualizing health promotion and health determinants Session on health care navigation

Low intention on health-promoting actions	Nutrition behavior (43.9%), stress management (39.5%), health responsible behavior (39.30%), social support (39.1%), life appreciative behavior (37.1%)	Limited concept of health-promoting actions and their domains	Session on conceptualizing health-promoting actions

Note. FGD = focus group discussion; HL = health literacy; HPE = health and physical education; ID = identifier for participant quote; KII = key informant interview.

### Low HL Awareness

Quantitative results showed significant gaps in basic HL awareness. Most adolescents have never heard about HL (71.3%), its meaning (74.7%), and have only limited awareness of the benefits of adequate HL and the consequences of inadequate HL. Qualitative insights confirmed these gaps, exploring a wider unfamiliarity with the HL notion. This gap highlights the need for an introductory session to raise the basic concept of HL.

### Inadequate Average HL

The quantitative findings reported that the participants' average HL levels were moderate (34.7%), and low (31.7%), indicating a prominent inadequate overall HL. Qualitative results similarly revealed that the participants' HL situation is entirely inadequate, reflecting their low self-efficacy in health information processing.

### Low Level Across HL Dimensions

Quantitative findings indicate that participants have low competence in the HL dimensions of health. Among the dimensions, accessing health information (40.7%) was the first need, followed by assessing (39.5%), applying (38.5%), and understanding (33.5%). In line with these needs, the qualitative stratum identified additional difficulties in adolescents' access to information, finding reliable sources, understanding information, making judgments, making informed health decisions, and using and sharing it. This result called for skill-based sessions to improve the HL situation.

### Difficulties in Health Domains

In the health domain, the quantitative study reported that health promotion was the main challenge for participants (37.7%), followed by health care (36.3%) and disease prevention. The qualitative study reported the same, additionally, expanding these results; they were entirely unfamiliar with the health promotion term, showed a medical-based understanding of health care, and had a unidimensional view of health and disease. These findings informed the need to include a broad conceptualization of health promotion, health, disease, its determinants, and health care navigation sessions.

### Low Intention on Health-Promoting Actions

The qualitative results indicated that adolescents' substantial gaps in engaging in health-promoting actions, including nutrition behavior (43.9%), stress management (39.5%), health responsible behavior (39.30%), social support (39.1%), and life appreciative behavior (37.1%). Qualitatively, these gaps were supported, underscoring the need for sessions focused on developing attitudes toward health-promoting actions.

The qualitative study further prioritized several critical areas, including substance use (such as drug use, smoking, and alcohol), low menstrual management, junk food consumption, and healthy eating habits; sexual and reproductive health topics, changes during adolescence; gang fights; and mental health. These gaps emphasize the need for targeted health literacy programs to meet these diverse needs.

### Preferred Key Contents, Learning Methods, Materials, Facilitators, and Modes of Administration

The qualitative results from multiple participants reported the contextual and preferred insights into facilitators, methods, and materials, and the mode of administration of the sessions for intervention implementation (**Table 4**). The involvement of school resources (health and physical education teachers and school nurses) and external resources (medical doctors, health education professors, and public health officers) was commonly preferred and recommended as facilitators. These findings informed the inclusion of expert-based and interdisciplinary facilitators that may enhance adolescents' motivations and participation.

**Table 4:** Facilitators, Methods/Materials, and Mode of Implementation of the Intervention Suggested by the Participants

**Description**	**Participants Responses**

Facilitators	

1. School resources such as the health teacher and the school nurse	All
2. External resources: doctors, health education experts, health education professors, and public health officers	All students, 2 HPE teachers and 2 nurses

Methods/materials	

1. Practical and interactive methods	All
2. Participation through discussions and activities focusing on students, audio-visual aids, PowerPoint, and figures	Few students, all teachers and nurses
3. Case studies, narrating experiences, stories, personal stories, and games	All teachers and a few nurses

Modes of implementation	

1. Classroom-based, in-person sessions	Most students, all teachers, and nurses
2. Online-based class	A few students and a nurse

Note. HPE = health and physical education.

Learner-centered, inductive, practical, and interactive lessons were recognized as a commonly accepted method across all participants, combined with interactive activities and materials. This was intended to imply a mixed teaching approach to deliver the intervention session. Classroom-based, in-person sessions within the school were commonly preferred and suggested as the mode of delivery, reflecting practicality and convenience for implementation.

### Delphi Consensus Results

In two rounds, consensus was reached on 52 items from different components of the intervention, including context (2 items), objective (3 items), contents (28 items), methods and materials (10 items), facilitators (3 items), duration (3 items), setting (1 item), and evaluation (2 items). In the first round, 41 items, and in the second round, 11 items received consensus, which were incorporated into the final intervention package.

### Setting Goals and Objectives of the Intervention

Based on the key gaps identified in the needs assessment phase, and further refined through a modified Delphi process, the intervention mainly aims to improve the overall HL of adolescents and their intentions to take health-promoting actions at the individual and peer levels. The researchers formulated the specific objectives linked to the key aim, contents, and the structure of the intervention. The objectives are aligned with the Comprehensive Model of HL, thus providing a theoretically and practically structured approach to improving the outcomes. Specific objectives included cognitive levels such as enhancing awareness, knowledge, attitudes, and practices of HL and health-promoting activities. Session-wise specific objectives are presented in detail in the intervention action plan (**Table A**).

**Table A:** Adolescent Health Literacy Intervention Action Plan

**SN**	**Objectives**	**Contents**	**Activities (SHOWED questions) and materials**	**Facilitators**	**Expected outcome**	**Evaluation**
1	Raise awareness of health concerns on HL	Adolescents' health concerns	Awareness raising activities on Health concerns through (example related to the low HL, showing photos related to miscommunication, experience sharing, tips and examples) and concept of health Literacy through story reading	Researcher	Identified various health concerns and problems,	Observation field note
		Concept of health literacy				
					Familiarized with HL terms	Quantitative and qualitative survey
2	Awareness and Confidence to demonstrate HL skills	Access, Understanding, Assess, Apply of health information	Demonstration activities related to seeking health information from the internet	Researcher	Developed confidence in HL skill	Observation field note
						Quantitative and qualitative survey
			PPT and a short example of a case study and real experiences related to assessing and using health information

**Table:** 

3	Society is a set of adolescent health	Social determinants of HL	Image showing related to determinants, watching a video regarding the barriers to using the health service	HPE teacher	Understood the multiple determinants of health and disease in their lives/ context	Observation field note
						Quantitative and qualitative survey
4	What should I communicate with the medical health service provider?	Health care	Activities to learn about health care, hospital, doctor, term, label, appointment, and medication	Medical doctor	Developed self-efficacy to practice medical care	Observation field note
		Communication with the service provider				
						Quantitative and qualitative survey
			Activities to recognize roles and functions of the provider through clear communication and role-playing			
5	In what way, prevent disease	Disease prevention: communicable, non-communicable, and mental illness	Activities to recognize the situation, risk factors and preventive measures of disease through facts, figures with plain communication and playing online game	School nurse	Increased KAP on prevention of disease.	Observation field note
						Quantitative and qualitative survey

**Table:** 

6	Promoting my health	Health promotion:	Plan language, Interaction and PPT with figure.	Researcher	Developed the concept and ways of health promotion	Observation field note
		Concept				
						Quantitative and qualitative survey
		Health-promoting actions				
7	To pursue healthy habit	Use of substances	Showing Video documentary related to smoking and experience sharing session.	Researcher	Improved understanding and motivation on threats, determinates and actions needed to eliminate smoking.	Observation field note
						Quantitative and qualitative survey
			Experienced shared by ex-druggist			
8	Intention to take healthy food and healthy eating	Nutrition behavior	Self-learning and story reading, picture (food pyramids), examples (eastern and western eating ways), ppt with nutritious information images.	Public health officer	Improved KAP on health food, eating and food label	Observation field note
						Quantitative and qualitative survey
9	Sensitize to move toward healthy habits	Health responsible behavior	Activities to recognize healthy and unhealthy behavior and its harms through snake ladder game.	HPE teacher	Improved self-awareness, self-efficacy to health	Observation field note
						Quantitative and

**Table:** 

					habits and their harms	qualitative survey
10	Develop KAP on dignified menstruation	Menstrual hygiene management, and Dignified menstruation	Experience sharing, and news reading	MPhil researcher	Developed KAP on dignified menstruation	Observation field note
						Quantitative and qualitative survey
11	Understanding about social support and stress management	Social support and stress management	Short role play of being blind, Relationship mapping and experience sharing related to stress, story reading	Social teacher	Increased social support. pursued stress management activities	Observation field note
						Quantitative and qualitative survey
12	Enthused toward life appreciating behavior	Life appreciating behavior	Mediation and reading research finding	Ayurveda Doctor	Understood life appreciating behavior	Observation field note
						Quantitative and qualitative survey
13	Develop self-efficacy for personal health actin plan	Set up a personal action plan	Activities related to skills learned in previous sessions to identify personal needs to develop action plan	Researcher	Pursued personal action plan	Observation field note qualitative survey

### Intervention Development

The study mapped the construction of a comprehensive HL model into conceptually focused intervention components, including four HL dimensions as outlined by Sørensen et al. (2012): accessing, understanding, appraising, and applying health information, and three health domains: health care, disease prevention, and health promotion. In this study, those were identified during a needs assessment phase and then refined through the Delphi process. Domains of health-promoting actions were incorporated into this intervention design to translate HL skills as an outcome application (**Table 5**). The theoretical mapping process supported selecting and organizing intervention materials, as well as creating an informed mix of identified needs, recommendations from experts, and theoretical knowledge (**Table 5**). The intervention components address single or multiple theoretical constructs are delivered using structured sessions detailed in the intervention action plan (**Table A**).

**Table 5:** Mapping Intervention Sessions to the Comprehensive Health Literacy Model Constructs

**Sessions Focused**	**Comprehensive Health Literacy Constructs**
Basic to adolescent health and health literacy	Understanding and expected outcome
Health literacy skills development	Self-efficacy, access, understanding, appraise, apply
Social determinants of health literacy	Understand and apply
Navigating the health service	Self-efficacy, access, understand, appraise, apply
Determinants and preventive measures of disease	Understand, appraise, apply
Basic concept of health promotion	Understand and appraise
Health-promoting actions	Self-efficacy, understand, appraise, and apply

Based on the Delphi findings, we drafted an initial version of the intervention and consulted with selected students, the HPE teacher, and the school nurse. The feedback obtained from discussions helped facilitate changes to ensure that the messages were reasonable and equally effective. To effectively promote HL, we finalized it in consultation with experts, and developed a detailed action plan (**Table A**).

### Contents Development

Mainly, all the contents of the intervention are tailored to the four dimensions of HL and three domains of health as proposed in the Comprehensive Model of HL and six constructs of the HPAs. The contents were tailored to address the specific needs identified during the needs assessment, aiming to empower students and peers to improve their HL knowledge, skills, and HPAs.

### Selection of Intervention Methods and Strategies

**Figure 2.**
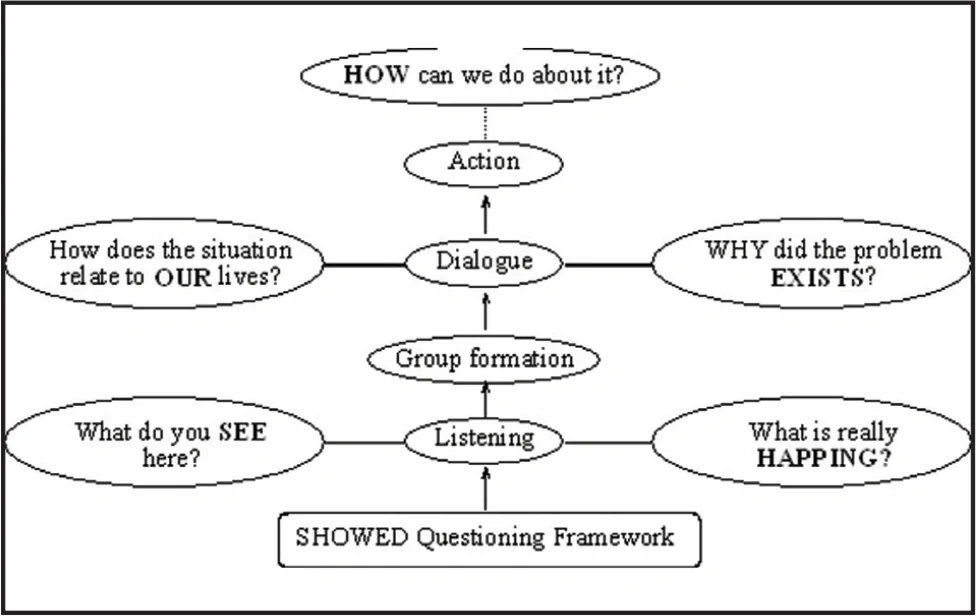
Intervention delivery methods and components.

Based on the participants' preferences as well as suggestions, the intervention design phase determined to employ art-based, interactive, and technological-based methods, promoting active learning and engaging students actively guided by the SHOWED Question framework as a dialogical method that fosters open dialogue, critical thinking, and personal engagement among students. This especially guided the delivery of intervention contents (**Figure 2**). Inductive teaching is an approach that encourages active participation and allows students to draw on their experiences and knowledge in the learning process.

### Resource Preparation and Selection

The development phase outlined the materials and resources to be developed and gathered to support implementation. These include art-based, interactive, and technological materials, such as PowerPoint presentations, handouts, stories, role-playing dialogues, pictures or posters, game materials, case studies, experience sharing, videos, documentaries, relationship mapping, news, and more.

### Implementation Structure Planning

This section reports on the intervention components constructed during the development phase. The elements described below in this structure reflect the intervention's delivery modality.

***Planning and preparation. ***During the intervention planning phase, an orientation meeting with potential facilitators was held to ensure a clear understanding of the intervention's thematic content, scope, ideas, delivery strategies, and duration, aligned with the established objectives. Such preparatory discussions were expected to align with the intervention's main goals, content, methods, materials, structure, modalities, and logistical arrangements before implementation. Contextual factors were consistently considered during the development phase so that the facilitators could tailor their roles to the intended methods, strategies, and materials within the specific context of the intervention, thereby enhancing relevance and feasibility. The design addressed all necessary logistical aspects, including scheduling sessions, preparing materials, and coordinating with school authorities to support effective delivery.

***Planned sessions. ***The final intervention package consists of 13 sessions: nine of approximately 1.5 hours and four of about 1 hour. A detailed action plan detailing session objectives, contents, methods, facilitators, expected output, and evaluation strategies was prepared as part of the intervention (**Table A**).

The development phase ensures that the intervention sessions are to be executed using the SHOWED questioning framework, structured into three key phases for each session: Listening, Dialogue, and Action. Each session is planned to begin with the listening phase (**Figure 2**), where the facilitator introduces the session's topic using interactive materials such as stories, games, or videos and PPT. Students are encouraged to observe, read, watch, and listen carefully, guided by initial questions like “What do you see here?” and “What is happening?” to prompt reflective understanding of the material presented.

The dialogue phase is planned to conduct small group discussions, guided by facilitators employing focused questions such as “How does the situation relate to our lives?” and “Why does the problem exist? This phase is expected to foster deep understanding and critical thinking by relating the session content to students' personal experiences and social contexts.

The action phase was designed to support translating the discussions to practical intentions and actions. Students are expected to jointly recognize actionable solutions regarding the session topic, guided by the question “What can we do about it?” Facilitators are expected to facilitate students develop actionable solutions and encourage the implementation of the knowledge they acquire.

***Facilitators selections and roles.*** In the intervention development phase, nine facilitators from interdisciplinary areas were selected to execute the particular session in a classroom setting, focusing on their expertise as informed by the needs assessment phase and the Delphi study and in order to ensure contextual appropriateness. To ensure effective and well-equipped delivery of the sessions, in-person orientation workshops were planned with a focus on a detailed review of the relevant session objectives, content, and on practical rehearsal of dialogical approaches and ethical considerations. To ensure consistency, it was planned to decide on and develop materials with the facilitator and provide him with a facilitator's manual. To maintain intervention fidelity, the principal investigator will monitor regular sessions, formative observation, support for the facilitators, and document the sessions' conduction process. This preparation effort is part of the intervention development and provides opportunities to collaboratively decide and prepare materials and develop a comprehensive plan to ensure the success of the program.

### Planned Evaluation Framework

The intervention development phase systematically planned an evaluation framework to inform the subsequent evaluation of the intervention's outcomes (**Table 6**). The primary outcome of the intervention will be to improve HL based on a comprehensive HL model. The secondary intended outcome will be to increase the intention to take health-promoting actions. The plan consisted of both formative and summative evaluation methods. Formative evaluation addressed qualitative FGDs, and KIIs at pre-posttest along with observational field notes and discussions with facilitators, school nurses, and students during implementation to ensure quality and fidelity. Summative evaluation incorporated pre-post-test standardized measures and qualitative methods immediately after the intervention to evaluate the intervention's outcomes.

**Table 6:** Planned Evaluation Methods and Tools

**Evaluation Types**	**Tools**	**Timing**	**Purpose**
Formative	Standardized quantitative scales and Qualitative FGDs and KIIs	Pre- post intervention	To identify HL needs, inform intervention design, and receive feedback regarding the intervention components
	Observational field notes, informal discussions	During the implementation	To receive the feedback for informing the delivery process (acceptability and feasibility)
Summative	Standardized quantitative scales and Qualitative FGDs and KIIs	Pre-post intervention	To determine the intervention's impact on the outcomes

Note. FGDs = focus group discussions; HL = health literacy; KIIs = key informant interviews.

## Discussion

This article presents the systematic development of a school-based HL intervention for adolescents, based on the comprehensive HL model (Sørensen et al., 2012) and HPAs (Chen et al., 2014), through mixed-methods needs analysis, and a modified Delphi study. The intervention mapping steps guided the development process (Bartholomew et al., 2006), tailoring the HL needs, stake-holders' views, and theory-driven strategies to design a context and concept-based intervention in the context of low-resource schools. Beyond a medical viewpoint, the context of intervention provides an organized approach developed to change multi-dimensional HL skills and HPAs among adolescents. Therefore, this study addresses the existing evidence gap in health knowledge and actions.

The clear theoretical mapping of intervention components into HL constructs and HPAs domains highlights significant achievement. Since there are some theory-based interventions such as physical activity-based programs (Knisel et al., 2020), problem-based learning HL intervention (Karimi et al., 2019), and social learning theory-informed intervention (Begjani et al., 2024), significantly focusing on limited health outcomes. However, the current intervention implies multidimensional outcomes, linking with the comprehensive HL model. The robust mapping method addresses the limitations of interventions that focus exclusively on functional HL (Visscher et al., 2018). This theory driven intervention is multidimensional and reliable, has clear conceptual clarity (French et al., 2012), and ensures effectiveness (Walters et al., 2020).

The intervention development was guided by an intervention mapping approach (Bartholomew et al., 2006) to align empirically identified multiple gaps, theoretical constructs, and implemented educational sessions. Rather than assuming global generalization, this intervention was designed to address contextual needs identified through a mixed-methods approach and literature review. The comprehensive needs-driven approach addresses gaps in the intervention's contextual relevance (French et al., 2012).

To deliver the learning sessions, the intervention design was linked to the SHOWED questioning framework, which is based on Freire's critical pedagogy (Freire, 2000). SHOWED advocates reflective actions such as listening, dialogue, and action to develop health HL skills. In the included schools, health education has traditionally been taught, and the initiation of a specific dialogue approach develops students' critical thinking using interactive and participatory learning actions (Meherali et al., 2020), without focusing on memorization. The application of mixed methods and dialogical approaches in intervention delivery may promote students' cognitive empowerment and critical HL skills. Evidence supported that such educational intervention based on a dialogical approach has the greatest influence on changing health behaviors (Jacobs et al., 2014).

The intervention's conceptual validity and contextual relevance were further strengthened by a two-stage modified Delphi study. Delphi methods have been reported to be used in diverse contexts of health education programs. Its use in the context of intervention development appears to be limited (Ravid-Saffir et al., 2023; Saxena et al., 2012) and developing scale (Bale et al., 2020; Roux et al., 2022), indicating limited applicability. In the present study, the interdisciplinary experts' consensus reinforced this intervention's components, the roles of facilitators, delivery strategy, and evaluation plan, thus strengthening the link between theory and practice. This ensured its evidence-based nature, validity, and reliability of the intervention (Roux et al., 2019), and heuristically integrated theoretical knowledge with contextual insights.

The design phase determined that the intervention sessions would be implemented in a school setting, integrating within the current school schedule of HPE and social studies subjects, as provided by the school, enhancing feasibility. This supports previous evidence that implementing school-based interventions during school hours is more effective than during additional hours (Durlak & DuPre, 2008). Coordination with schools will be prioritized in scheduling each session, as schools prioritize conducting exams and completing courses. This will support reducing implementation barriers (Schultes, 2023).

The intervention development phase outlined that the distribution of the facilitators' workload based on the interdisciplinary specialization within the school, such as school HPE and social teacher, school nurse, and beyond the school, including health education educators, medical doctors, and public health officers. This may balance the facilitator and ensure ownership, valuing the fidelity and long-lastingness of the intervention (Fixsen et al., 2005). The intervention design outlined the organized manual and session plans, providing further support for effective implementation across the intervention group (Schultes, 2023). Being based on low-resource and technological facilities, the low-resource context is flexible, and it has the potential for school-based intervention adaptation from pilot trials to broader settings, thus, it is possible to scale up within the same school context.

Several limitations have to be considered. Although school stakeholders were involved, parents remained involved in the process. This may determine the intention of HPAs beyond the schools. The curriculum-based content review has not yet been performed robustly. Although the needs assessment study suggested multiple determinants of HL, due to practical limitations, this intervention was fundamentally designed focusing only on the individual and peer level. The sampling method could have been biased, due to focus group discussions, the social desirability bias might have been present. This suggests that the results should be viewed carefully when it comes to generalizability and transferability. Moreover, the intervention was based on the SHOWED approach, and limited student participation remained in the materials and methods decisions. The availability of facilitators for implementing the SHOWED approach is difficult, and the time allocated for some of the content in the intervention was slightly less. The technological activities that students preferred were limited to being included in the intervention delivery, due to the researcher's and context's limitations. Finally, this article, limited to the intervention design, remains to empirically test the outcome evaluation and sustainability of the intervention.

Finally, this intervention design differs from previous studies in several ways. This intervention design differs from previous studies in several ways. It is theory-driven, based on an intervention mapping approach, and further refined through the Delphi process. Employing dialogical pedagogy, this intervention design introduces organized educational strategies for HL skill development. Fidelity, effectiveness, and scalability of implementation should be considered in future searches to determine their greater relevance to adolescent health promotion. This intervention design can also be adapted for adults, families, and multilevel settings.

## Conclusion

This intervention is designed using an intervention mapping approach tailored to the EU HL model. A modified Delphi study strengthens its validity. The intervention directly aligns with the AHL needs, targeting improvements in HL and HPAs at both the individual and peer levels. It consists of 13 sessions led by interdisciplinary facilitators using the SHOWED questioning framework. Overall, the study emphasizes the value of a thorough, theory-based, context-specific strategy for raising AHL and encouraging HPAs. With further adaptations, it has the potential to be used in a different setting.

## References

[x24748307-20260413-01-bibr1] Bale , J. , Grové , C. , & Costello , S. ( 2020 ). Building a mental health literacy model and verbal scale for children: Results of a Delphi study . *Children and Youth Services Review* , *109* , 104667 . 10.1016/j.childyouth.2019.104667

[x24748307-20260413-01-bibr2] Bartholomew , L. K. , Parcel , G. S. , Kok , G. , & Gottlieb , N. H. ( 2006 ). *Planning health promotion programs: An intervention mapping approach* ( 2nd ed. ). Jossey-Bass .

[x24748307-20260413-01-bibr3] Baumeister , A. , Chakraverty , D. , Aldin , A. , Seven , Ü. S. , Skoetz , N. , Kalbe , E. , & Woopen , C. ( 2021 ). “The system has to be health literate, too” - perspectives among healthcare professionals on health literacy in transcultural treatment settings . *BMC Health Services Research* , *21* ( 1 ), 716 . 10.1186/s12913-021-06614-x PMID: 34289853 PMC8293586

[x24748307-20260413-01-bibr4] Begjani , J. , Hosseini , A. S. S. , Saneifard , H. , & Hasanabad , V. R. ( 2024 ). Social learning-based health literacy promotion on the self efficacy and social anxiety of adolescents with type 1 diabetes . *Clinical Diabetes and Endocrinology* , *10* ( 1 ), 14 . 10.1186/s40842-024-00167-8 PMID: 38486333 PMC10941360

[x24748307-20260413-01-bibr5] Bhatta , B. K. , Tiwari , R. , Ghimire , S. , Khadka , R. , & Malasi , A. ( 2024 ). Nutritional status and its associated factors among government school-going adolescents of Birendranagar Municipality, Surkhet, Nepal . *Nepal Medical College Journal* , *26* ( 2 ), 150 – 156 . 10.3126/nmcj.v26i2.67211

[x24748307-20260413-01-bibr6] Bollweg , T. M. , Okan , O. , Freţian , A. M. , Bröder , J. , Domanska , O. M. , Jordan , S. , Bruland , D. , Pinheiro , P. , & Bauer , U. ( 2020 ). Adapting the European Health Literacy Survey Questionnaire for fourth-grade students in Germany: Validation and psychometric analysis . *Health Literacy Research and Practice* , *4* ( 3 ), e144 – e159 . 10.3928/24748307-20200428-01 PMID: 32674162 PMC7365660

[x24748307-20260413-01-bibr7] Bollweg , T. M. , Okan , O. , Pinheiro , P. , Bröder , J. , Bruland , D. , Freţian , A. M. , Domanska , O. M. , Jordan , S. , & Bauer , U. ( 2020 ). Adapting the European Health Literacy Survey for fourth-grade students in Germany: Questionnaire Development and qualitative pretest . *Health Literacy Research and Practice* , *4* ( 2 ), e119 – e128 . 10.3928/24748307-20200326-01 PMID: 32392350 PMC7213025

[x24748307-20260413-01-bibr8] Bröder , J. , Okan , O. , Bauer , U. , Bruland , D. , Schlupp , S. , Bollweg , T. M. , Saboga-Nunes , L. , Bond , E. , Sørensen , K. , Bitzer , E.-M. , Jordan , S. , Domanska , O. , Firnges , C. , Carvalho , G. S. , Bittlingmayer , U. H. , Levin-Zamir , D. , Pelikan , J. , Sahrai , D. , Lenz , A. , Pinheiro , P. ( 2017 ). Health literacy in childhood and youth: A systematic review of definitions and models . *BMC Public Health* , *17* ( 1 ), 361 . 10.1186/s12889-017-4267-y PMID: 28441934 PMC5405535

[x24748307-20260413-01-bibr9] Casteleijn , M . ( 2016 ). *Holistic Health Literacy- Moving (Youth) Health Literacy Upstream* Wageningen University]. Bridge for Health Literacy Project . https://edepot.wur.nl/394474

[x24748307-20260413-01-bibr10] Chen , M.-Y. , Lai , L.-J. , Chen , H.-C. , & Gaete , J. ( 2014 ). Development and validation of the short-form adolescent health promotion scale . *BMC Public Health* , *14* ( 1 ), 1106 . 10.1186/1471-2458-14-1106 PMID: 25344693 PMC4216378

[x24748307-20260413-01-bibr11] Cresswell , J. W. ( 2012 ). *Educational research: Planning, conducting and evaluating quantitative and qualitative research* ( 4th ed. ). Pearson Education Inc .

[x24748307-20260413-01-bibr12] Creswell , J. W. , & Miller , D. L. ( 2000 ). Determining validity in qualitative inquiry . *Theory Into Practice* , *39* ( 3 ), 124 – 130 . 10.1207/s15430421tip3903_2

[x24748307-20260413-01-bibr13] Creswell , J. W. , & Creswell , J. D. ( 2018 ). *Research design: Qualitative, quantitative and mixed methods approaches* ( 5th ed. ). SAGE Publications, Inc .

[x24748307-20260413-01-bibr14] Dhimal , M. , Dahal , S. , Adhikari , K. , Koirala , P. , Bista , B. , Luitel , N. , Pant , S. , Marahatta , K. , Shakya , S. , Sharma , P. , Ghimire , S. , Gyanwali , P. , Ojha , S. P. , & Jha , A. K. ( 2022 ). A nationwide prevalence of common mental disorders and suicidality in Nepal: Evidence from National Mental Health Survey, 2019–2020 . *J Nepal Health Res Counc* , *19* ( 4 ), 740 – 747 . doi: 10.33314/jnhrc.v19i04.4017 PMID: 35615831

[x24748307-20260413-01-bibr15] Durlak , J. A. , & DuPre , E. P. ( 2008 ). Implementation matters: A review of research on the influence of implementation on program outcomes and the factors affecting implementation . *American Journal of Community Psychology* , *41* ( 3–4 ), 327 – 350 . 10.1007/s10464-008-9165-0 PMID: 18322790

[x24748307-20260413-01-bibr16] Fixsen , D. L. , Naoom , S. F. , Blase , K. A. , Friedman , R. M. , & Wallace , F . ( 2005 ). *Implementation research: A synthesis of the literature (FMHI Publication #231)* . Tampa, FL : University of South Florida, Louis de la Parte Florida Mental Health Institute, The National Implementation Research Network . https://nirn.fpg.unc.edu/wp-content/uploads/NIRN-MonographFull-01-2005.pdf

[x24748307-20260413-01-bibr17] Freire , P . ( 2000 ). *Pedagogy of the oppressed* ( 30th anniversary ed. ). Continuum .

[x24748307-20260413-01-bibr18] French , S. D. , Green , S. E. , O'Connor , D. A. , McKenzie , J. E. , Francis , J. J. , Michie , S. , Buchbinder , R. , Schattner , P. , Spike , N. , & Grimshaw , J. M. ( 2012 ). Developing theory-informed behaviour change interventions to implement evidence into practice: A systematic approach using the Theoretical Domains Framework . *Implementation Science* , *7* ( 1 ), 38 . 10.1186/1748-5908-7-38 22531013 PMC3443064

[x24748307-20260413-01-bibr19] Gordon , S. C. , Barry , C. D. , Dunn , D. J. , & King , B. ( 2011 ). Clarifying a vision for health literacy: A holistic school-based community approach . *Holistic Nursing Practice* , *25* ( 3 ), 120 – 126 . doi: 10.1097/hnp.0b013e3182157c34 PMID: 21508711

[x24748307-20260413-01-bibr20] Jacobs , R. J. , Lou , J. Q. , Ownby , R. L. , & Caballero , J. ( 2014 ). A systematic review of eHealth interventions to improve health literacy . *Health Informatics Journal* , *22* ( 2 ), 81 – 98 . 10.1177/1460458214534092 24916567

[x24748307-20260413-01-bibr21] Kanj , M. , & Mitic , W . ( 2009, October 26–30 ). *Health literacy and health promotion: Definitions, concepts and examples in the Eastern Mediterranean region* [Document for discussion]. 7th Global Conference on Health Promotion, “Promoting Health and Development: Closing the Implementation Gap” , Nairobi, Kenya .

[x24748307-20260413-01-bibr22] Karimi , N. , Saadat-Gharin , S. , Tol , A. , Sadeghi , R. , Yaseri , M. , & Mohebbi , B. ( 2019 ). A problem-based learning health literacy intervention program on improving health-promoting behaviors among girl students . *Journal of Education and Health Promotion* , *8* , 251 . 10.4103/jehp.jehp_476_19 32002423 PMC6967126

[x24748307-20260413-01-bibr23] Khanal , S. P. , Budhathoki , C. B. , Okan , O. , van Teijlingen , E. , Sharma , M. K. , Acharya , J. , & Wood , C. ( 2023 ). Systematic review of health literacy and health promotion in school-aged adolescents . *Journal of Education and Community Health* , *10* ( 1 ), 49 – 57 . doi: 10.34172/jech.2023.1982

[x24748307-20260413-01-bibr24] Khanal , S. P. , Budhathoki , C. B. , Devkota , B. , Bollweg , T. M. , & Okan , O. ( 2024 ). Adolescents' understanding of the Nepalese version of HLS-CHILD-Q15: Qualitative pre-testing in ninth-graders . *BMC Public Health* , *24* ( 1 ), 851 . 10.1186/s12889-024-18329-9 PMID: 38504195 PMC10949603

[x24748307-20260413-01-bibr25] Khanal , S. P. , Budhathoki , C. B. , & Okan , O. ( 2023 ). Improving adolescent health literacy through school-based health literacy intervention: A mixed-method study protocol . *BMC Public Health* , *23* ( 1 ), 407 . 10.1186/s12889-023-15316-4 PMID: 36855125 PMC9973246

[x24748307-20260413-01-bibr26] Khanal , S. P. , Budhathoki , C. B. , & Okan , O. ( 2025 ). Effectiveness of a school-based health literacy intervention in improving adolescent health literacy and the intention to take health-promoting actions . *BMC Public Health* , *25* ( 1 ), 3551 . 10.1186/s12889-025-24827-1 PMID: 41121137 PMC12542496

[x24748307-20260413-01-bibr27] Khanal , S. P. , Budhathoki , C. B. , Sharma , M. K. , & Okan , O. ( 2026 ). Assessing the health literacy needs of school adolescents in Birendranagar Municipality, Surkhet, Nepal . *BMC Public Health* , *26* ( 1 ), 433 . 10.1186/s12889-025-26084-8 PMID: 41485008 PMC12866528

[x24748307-20260413-01-bibr28] Khanal , S. P. , Sharma , M. K. , Kharel , S. , & Sharma , C. ( 2021 ). Role of health literacy on health behaviour change among the college girl students . *Scholars' Journal* , *4* ( 1 ), 179 – 189 . 10.3126/scholars.v4i1.42478

[x24748307-20260413-01-bibr29] Kirchhoff , S. , Dadaczynski , K. , Pelikan , J. M. , Zelinka-Roitner , I. , Dietscher , C. , Bittlingmayer , U. H. , & Okan , O. ( 2022 ). Organizational health literacy in schools: Concept development for health-literate schools . *International Journal of Environmental Research and Public Health* , *19* ( 14 ), 8795 . 10.3390/ijerph19148795 PMID: 35886647 PMC9316432

[x24748307-20260413-01-bibr30] Kirchhoff , S. , Krudewig , C. , & Okan , O . ( 2025 ). Organizational health literacy of schools in Germany: Results of a cross-sectional study . *Health Promotion International* , *40* ( 4 ), daaf112 . 10.1093/heapro/daaf112 PMID: 40754692 PMC12318714

[x24748307-20260413-01-bibr31] Knisel , E. , Rupprich , H. , Wunram , A. , Bremer , M. , & Desaive , C. ( 2020 ). Promotion of elementary school students' health literacy . *International Journal of Environmental Research and Public Health* , *17* ( 24 ), 9560 . 10.3390/ijerph17249560 PMID: 33371224 PMC7766722

[x24748307-20260413-01-bibr32] Lincoln , Y. S. , & Guba , E. G. ( 1985 ). *Naturalistic inquiry* . Beverly Hills, CA : Sage Publications .

[x24748307-20260413-01-bibr33] Locke , K. , Feldman , M. , & Golden-Biddle , K. ( 2022 ). Coding practices and iterativity: Beyond templates for analyzing qualitative data . *Organizational Research Methods* , *25* ( 2 ), 262 – 284 . 10.1177/1094428120948600

[x24748307-20260413-01-bibr34] Massey , P. M. , Prelip , M. , Calimlim , B. M. , Quiter , E. S. , & Glik , D. C. ( 2012 ). Contextualizing an expanded definition of health literacy among adolescents in the health care setting . *Health Education Research* , *27* ( 6 ), 961 – 974 . 10.1093/her/cys054 PMID: 22623619 PMC3498601

[x24748307-20260413-01-bibr35] McKenna , V. B. , Mathew , J. B. , Finn , Y. , & Sixsmith , J . ( 2025 ). Approaches to assessment of community level health literacy: A scoping review . *Health Promotion International* , *40* ( 5 ), daaf123 . 10.1093/heapro/daaf123 PMID: 41124601 PMC12543020

[x24748307-20260413-01-bibr36] Meherali , S. , Punjani , N. S. , & Mevawala , A. ( 2020 ). Health literacy interventions to improve health outcomes in low- and middle-income countries . *Health Literacy Research and Practice* , *4* ( 4 ), e251 – e266 . doi: 10.3928/24748307-20201118-01 PMID: 33313935 PMC7751448

[x24748307-20260413-01-bibr37] Ministry of Health and Population (Nepal), New ERA, & ICF . ( 2022 , November ). *Nepal demographic and health survey 2022: Key indicators report* . Kathmandu, Nepal : MOHP . https://dhsprogram.com/pubs/pdf/PR142/PR142.pdf

[x24748307-20260413-01-bibr38] Morgado , T. M. M. , Loureiro , L. M. J. , & Rebelo Botelho , M. A. M. ( 2022 ). Psychoeducational interventions to promote adolescents' mental health literacy in schools: Identifying theory for the development of a complex intervention . *Journal of Child and Adolescent Psychiatric Nursing: Official Publication of the Association of Child and Adolescent Psychiatric Nurses, Inc* . , *35* ( 4 ), 331 – 340 . 10.1111/jcap.12386 PMID: 35748243

[x24748307-20260413-01-bibr39] Nash , R. , Patterson , K. , Flittner , A. , Elmer , S. , & Osborne , R. ( 2021 ). School-based health literacy programs for children (2–16 years): An international review . *The Journal of School Health* , *91* ( 8 ), 632 – 649 . 10.1111/josh.13054 PMID: 34096058

[x24748307-20260413-01-bibr40] Nastasi , B. K. , Hitchcock , J. , Sarkar , S. , Burkholder , G. , Varjas , K. , & Jayasena , A. ( 2007 ). Mixed methods in intervention research: Theory to adaptation . *Journal of Mixed Methods Research* , *1* ( 2 ), 164 – 182 . 10.1177/1558689806298181

[x24748307-20260413-01-bibr41] Okan , O. , Paakkari , L. , & Dadaczynski , K . ( 2020 ). *Health literacy in schools. State of the art* . Schools for Health in Europe Network Foundation . https://www.schoolsforhealth.org/sites/default/files/editor/fact-sheets/factsheet-2020-english.pdf

[x24748307-20260413-01-bibr42] Rauschmayr , S. , Krudewig , C. , & Okan , O . ( 2025 ). *Health-literate pre-schools: A guide for early childhood education institutions* . Technical University of Munich . 10.14459/2025md1774240

[x24748307-20260413-01-bibr43] Ravid-Saffir , A. , Sella , S. , & Ben-Eli , H. ( 2023 ). Development and validation of a questionnaire for assessing parents' health literacy regarding vision screening for children: A Delphi study . *Scientific Reports* , *13* ( 1 ), 13887 . 10.1038/s41598-023-41006-7 37620666 PMC10449776

[x24748307-20260413-01-bibr44] Riiser , K. , Helseth , S. , Haraldstad , K. , Torbjørnsen , A. , & Richardsen , K. R. ( 2020 ). Adolescents' health literacy, health protective measures, and health-related quality of life during the Covid-19 pandemic . *PLoS One* , *15* ( 8 ), e0238161 . 10.1371/journal.pone.0238161 PMID: 32857806 PMC7454983

[x24748307-20260413-01-bibr45] Ritchie , J. , & Spencer , L . ( 1994 ). Qualitative data analysis for applied policy research . In Bryman A. & Burgess R. (Eds.), *Analyzing Qualalitative Data* (pp. 173 – 194 ). Routledge . 10.4324/9780203413081_chapter_9

[x24748307-20260413-01-bibr46] Roux , F. , Burns , S. , Chih , H. J. , & Hendriks , J. ( 2019 ). Developing and trialling a school-based ovulatory-menstrual health literacy programme for adolescent girls: A quasi-experimental mixed-method protocol . *BMJ Open* , *9* ( 3 ), e023582 . 10.1136/bmjopen-2018-023582 PMID: 30898802 PMC6528013

[x24748307-20260413-01-bibr47] Roux , F. , Burns , S. , Chih , H. , & Hendriks , J . ( 2022 ). The use of a two-phase online Delphi panel methodology to inform the concurrent development of a school-based ovulatory menstrual health literacy intervention and questionnaire [Brief Research Report] . *Frontiers in Global Women's Health* , *3* . 10.3389/fgwh.2022.826805 PMC9168325 35677755

[x24748307-20260413-01-bibr48] Santana , S. , Brach , C. , Harris , L. , Ochiai , E. , Blakey , C. , Bevington , F. , Kleinman , D. , & Pronk , N. ( 2021 ). Updating health literacy for healthy people 2030: Defining its importance for a new decade in public health . *Journal of Public Health Management and Practice* , *27* ( Suppl. 6 ), S258 – S264 . 10.1097/PHH.0000000000001324 33729194 PMC8435055

[x24748307-20260413-01-bibr49] Sapkota , S. , Chaudhari , A. , Rawal , P. , Paudel , K. N. , & Giri , P. ( 2024 ). Knowledge and perspective regarding adolescent sexual and reproductive health among adolescents in selected schools of Birendranagar Mucinipality of Surkhet, Karnali Province . *NPRC Journal of Multidisciplinary Research* , *1* ( 4 ), 134 – 158 . 10.3126/nprcjmr.v1i4.70959

[x24748307-20260413-01-bibr50] Saxena , D. , Kumar , P. , Rana , M. , & Shah , H. ( 2012 ). A case study on use of modified Delphi technique for developing consensus on designing contents of a module for imparting sex education to adolescents in schools, in India . *Global Journal of Medicine and Public Health* , *1* , 3 – 7 .

[x24748307-20260413-01-bibr51] Schneider , P. , Evaniew , N. , Rendon , J. S. , McKay , P. , Randall , R. L. , Turcotte , R. , Vélez , R. , Bhandari , M. , Ghert , M. , & the PARITY Investigators . ( 2016 ). Moving forward through consensus: Protocol for a modified Delphi approach to determine the top research priorities in the field of orthopaedic oncology . *BMJ Open* , *6* ( 5 ), e011780 . 10.1136/bmjopen-2016-011780 PMID: 27221129 PMC4885431

[x24748307-20260413-01-bibr52] Schultes , M.-T. ( 2023 ). An introduction to implementation evaluation of school-based interventions . *European Journal of Developmental Psychology* , *20* ( 1 ), 189 – 201 . 10.1080/17405629.2021.1976633

[x24748307-20260413-01-bibr53] Schwarzer , R. , & Jerusalem , M . ( 1995 ). Generalized Self-Efficacy scale . In Weinman J. , Wright S. , & Johnston M. (Eds.), *Measures in health psychology: A user's portfolio. Causal and control beliefs* (pp. 35 – 37 ). Windsor, UK : NFER-NELSON . https://userpage.fu-berlin.de/%7Ehealth/engscal.htm

[x24748307-20260413-01-bibr54] Sørensen , K. , Van den Broucke , S. , Fullam , J. , Doyle , G. , Pelikan , J. , Slonska , Z. , Brand , H. , & the (HLS-EU) Consortium Health Literacy Project European . ( 2012 ). Health literacy and public health: A systematic review and integration of definitions and models . *BMC Public Health* , *12* , 80 . 10.1186/1471-2458-12-80 PMID: 22276600 PMC3292515

[x24748307-20260413-01-bibr55] Visscher , B. B. , Steunenberg , B. , Heijmans , M. , Hofstede , J. M. , Devillé , W. , van der Heide , I. , & Rademakers , J. ( 2018 ). Evidence on the effectiveness of health literacy interventions in the EU: A systematic review . *BMC Public Health* , *18* , 1414 . 10.1186/s12889-018-6331-7 30594180 PMC6310940

[x24748307-20260413-01-bibr56] Walters , R. , Leslie , S. J. , Polson , R. , Cusack , T. , & Gorely , T. ( 2020 ). Establishing the efficacy of interventions to improve health literacy and health behaviours: A systematic review . *BMC Public Health* , *20* ( 1 ), 1040 . 10.1186/s12889-020-08991-0 PMID: 32605608 PMC7329558

[x24748307-20260413-01-bibr57] World Health Organization (WHO) . ( 2017 ). *Shanghai declaration on promoting health in the 2030 Agenda for Sustainable Development* . https://www.who.int/publications/i/item/WHO-NMH-PND-17.5 10.1093/heapro/daw103 28180270

[x24748307-20260413-01-bibr58] Wilson , N. , Dasho , S. , Martin , A. , Wallerstein , N. , Wang , C. , & Minkler , M. ( 2007 ). Engaging young adolescents in social action through photovoice: The Youth Empowerment Strategies (YES!) Project . *Journal of Early Adolescence* , *27* ( 2 ), 241 – 261 . 10.1177/0272431606294834

